# Characterization of a Citrus R2R3-MYB Transcription Factor that Regulates the Flavonol and Hydroxycinnamic Acid Biosynthesis

**DOI:** 10.1038/srep25352

**Published:** 2016-05-10

**Authors:** Chaoyang Liu, Jianmei Long, Kaijie Zhu, Linlin Liu, Wei Yang, Hongyan Zhang, Li Li, Qiang Xu, Xiuxin Deng

**Affiliations:** 1 Key Laboratory of Horticultural Plant Biology (Ministry of Education), Huazhong Agricultural University, Wuhan 430070, P. R. China; 2 Robert W. Holley Center for Agriculture and Health, USDA-ARS, Plant Breeding and Genetics Section, School of Integrative Plant Science, Cornell University, Ithaca, NY 14853, USA

## Abstract

Flavonols and hydroxycinnamic acids are important phenylpropanoid metabolites in plants. In this study, we isolated and characterized a citrus R2R3-MYB transcription factor *CsMYBF1*, encoding a protein belonging to the flavonol-specific MYB subgroup. Ectopic expression of *CsMYBF1* in tomato led to an up-regulation of a series of genes involved in primary metabolism and the phenylpropanoid pathway, and induced a strong accumulation of hydroxycinnamic acid compounds but not the flavonols. The RNAi suppression of *CsMYBF1* in citrus callus caused a down-regulation of many phenylpropanoid pathway genes and reduced the contents of hydroxycinnamic acids and flavonols. Transactivation assays indicated that CsMYBF1 activated several promoters of phenylpropanoid pathway genes in tomato and citrus. Interestingly, CsMYBF1 could activate the *CHS* gene promoter in citrus, but not in tomato. Further examinations revealed that the MYBPLANT cis-elements were essential for CsMYBF1 in activating phenylpropanoid pathway genes. In summary, our data indicated that CsMYBF1 possessed the function in controlling the flavonol and hydroxycinnamic acid biosynthesis, and the regulatory differences in the target metabolite accumulation between two species may be due to the differential activation of *CHS* promoters by CsMYBF1. Therefore, *CsMYBF1* constitutes an important gene source for the engineering of specific phenylpropanoid components.

Phenylpropanoids constitute a major group of secondary metabolites and have various biological functions, such as UV protection, structural polymers, signal compounds, defense response, pigments and attractants for pollinators^1^,^2^. Flavonols, the ancient and widespread class of phenylpropanoids, exhibit great antioxidant potential and are thought to be effective UV filters, thereby perform important functional roles during plant evolution^3^. Flavonols also exhibit anti-inflammatory and anti-proliferative capacities and have great beneficial effects on human health including protection against cancer and cardiovascular disease^4^. Hydroxycinnamic acids, the major subgroup of phenolic acids, are the important source of antioxidants due to their widely occurrence in plants and the powerful radical scavenging activities^5^.

The biosynthetic pathway of phenolic compounds in plants has been well elucidated in many plant species^6^,^7^. The plant shikimate pathway, which connects central carbon metabolism and the aromatic amino acid pathways, is the entry to the biosynthesis of phenylpropanoids. The enzymes catalyzing the initial steps of the phenylpropanoid pathway are PAL, C4H, and 4CL (Fig. 1). These three steps are necessary for the biosynthesis of hydroxycinnamic acids and their derivatives, which are precursors for all the other types of phenylpropanoids^6^. The first committed step of flavonoid biosynthesis is catalysed by CHS, which is known as the gatekeeper of flavonoid biosynthesis and plays an important role in regulating the pathway^8^. The initial product of CHS is further converted to other flavonoid classes, such as flavonols, flavones, flavanones and anthocyanidins. Specially, FLS is responsible for the synthesis of flavonols (Fig. 1).

Accumulating evidence indicate that the phenylpropanoid pathway genes are predominantly regulated by the R2R3-MYB transcription factors. Recently, a number of MYB regulators of flavonol biosynthesis have been identified. In grapevine, the expression pattern of *VvMYBF1* directly correlates with that of *VvFLS1* and subsequent flavonol accumulation^9^. In Japanese gentian (*Gentiana triflora*), GtMYBP3 and GtMYBP4 activate the expression of flavonol biosynthesis genes and increase flavonol accumulation when heterologously expressed in tobacco and *Arabidopsis*^10^. It is noteworthy that the characterized flavonol-related MYB proteins are usually involved in the regulation of the other branches of phenylpropanoid pathway, particularly the hydroxycinnamic acid metabolism. AtMYB12, the flavonol-specific transcriptional activator in *Arabidopsis*, induces the accumulation of high levels of caffeoyl quinic acids and flavonols when overexpressed in tomato and tobacco^11^,^12^. AtMYB11 and AtMYB111, the closest homologs of AtMYB12 in *Arabidopsis*, also show similar functions when overexpressed in heterologous systems^13^,^14^. In maize, the flavonol regulator *P1*, could affect the levels of specific phenylpropanoids such as ferulic acid when overexpressed in cultured maize cells, and the corresponding p1 locus is also implicated in controlling the levels of chlorogenic acid in maize silk tissue^15^,^16^,^17^,^18^.

Some MYB transcription factors involved in the regulation of hydroxycinnamic acid biosynthesis have also been characterized. For instance, AtMYB4 in Arabidopsis, VvMYB4a and VvMYB4b in grapevine, are characterized as negative regulators of hydroxycinnamic acid metabolism^19^,^20^. Several MYB regulators of hydroxycinnamic acid biosynthesis are also involved in the regulation of other classes of phenylpropanoid pathway, such as the VvMYBC2-L1 and VvMYBC2-L3 that additionally control the flavonoid levels in grapevine^20^. Increasing evidence reveal that many MYB transcription factors could impact on various branches of phenylpropanoid metabolism simultaneously, and even regulate nearly the whole pathway in some cases^21^, which may reflect the elaborate and complex transcriptional regulation mechanisms in plants.

Citrus is one of the most important fruit crops worldwide and citrus plants are abundant with phenolic compounds. Citrus plants contain various kinds of esters and glycosides of hydroxycinnamic acids, which carried out important functions in plant defense and fruit maturation processes^22^,^23^. The spatiotemporal accumulation of flavonols is divergent in citrus organs. The flavonol compounds were preferentially accumulated in leaves of most citrus varieties, while only a very small amount of flavonols was found in fruit tissues of a limited number of varieties such as lemon and lime^24^,^25^. The tissue-specific accumulation pattern implicates the special regulation mechanism of flavonol biosynthesis in citrus.

Recently, increasing insights on the biochemical properties of the phenolic compounds have been gained in citrus. However, our knowledge about the molecular mechanism underlying the biosynthesis and regulation of the phenylpropanoid metabolism is limited. Some structural genes of the phenylpropanoid pathway in citrus, like CHS and FLS genes, were isolated and their expression patterns have been investigated. The high transcript levels of citrus *FLS* gene in young leaves and the early developmental stage of the pulp tissues indicated that this gene was differentially regulated in the developmental stage and in a tissue-specific manner^26^. For the regulation of the structural genes, only the CsRuby, a R2R3-MYB transcription factor, was characterized as the anthocyanin regulator in blood orange, implicating the important roles of citrus MYB transcription factors in phenylpropanoid metabolism regulation^27^.

A total of 100 R2R3-MYB transcription factors have been identified in the genome of sweet orange, the principal variety of citrus species. Further phylogenetic analysis identified several putative MYB functional clades that were involved in phenylpropanoid metabolism including lignins, anthocyanins and flavonols biosynthesis^28^. Due to the importance of flavonols in plant physiology and human health and their differential accumulation pattern across citrus tissues, the flavonol-related MYB subgroup was selected for further investigation. In the present study, the citrus R2R3-MYB transcription factor *CsMYBF1* was isolated. Functional analysis including ectopic expression in tomato and the RNAi suppression in citrus callus indicated that *CsMYBF1* was involved in the regulation of the flavonol and hydroxycinnamic acid biosynthesis. Interestingly, overexpression of *CsMYBF1* in tomato fruits reduced the contents of the major sugars and specially increased the amount of hydroxycinnamic acid compounds but not the flavonols. The unexpected phenotype changes were found to be due to the differential activation of *CHS* promoters by CsMYBF1, some potential factors including variation in MYBPLANT cis-elements may affect such regulatory differences.

## Results

### Sequence features of CsMYBF1

**Figure 2:**
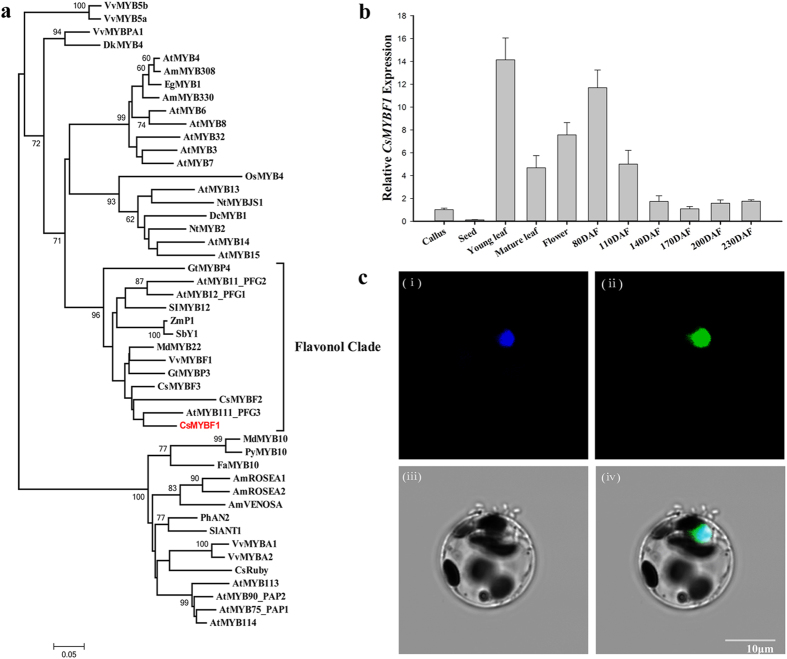
Sequence comparison, expression pattern and subcellular localization of CsMYBF1. (**a**) Phylogenetic analysis of selected plant MYB proteins. The scale bar represents 0.05 substitutions per site. GenBank accession numbers of the proteins are listed in Supplementary Table S1. (**b**) Expression pattern of *CsMYBF1* in different tissues of citrus. Data are means ± SE of three replicate PCRs. (**c**) The subcellular localization of CsMYBF1 in citrus protoplast. OsGhd7-CFP and CsMYBF1-GFP were co-transformed into citrus mesophyll protoplasts. OsGhd7-CFP was used as a nuclear marker. (i) OsGhd7-CFP , (ii) CsMYBF1-GFP, (iii) bright field, (iv) merged picture.

Three putative flavonol-related MYB transcription factors were identified from the genome-wide analysis of the sweet orange genome^28^, and these proteins were named as CsMYBF1 (Cs9g02640), CsMYBF2 (Cs5g33870) and CsMYBF3 (Cs5g33880), respectively. CsMYBF1 was selected for further analysis in this study, based on its expression feature and transactivation ability. The full-length of *CsMYBF1* cDNA from sweet orange was obtained by 5′/3′ RACE experiments (GeneBank accession number KT727073). Analysis of the deduced amino acid sequence revealed that CsMYBF1 contained the R2R3 imperfect repeats involved in binding to target DNA sequences and highly conserved among MYB proteins. Apart from the significant sequence similarity among the MYB proteins within the N-terminus, the redefined SG7 motif ([K/R][R/x][R/K]xGRT[S/x][R/G]xx[M/x]K) and the SG7-2 motif ([W/x][L/x]LS)^7^, which were characteristic of flavonol regulators, were partially conserved in CsMYBF1 (Supplementary Figure S1). A phylogenetic analysis of 48 MYB domains by the neighbor-joining method indicated that CsMYBF1 belonged to the flavonol clade which consisting of the MYB flavonol regulators from various plant species (Fig. 2a). The protein sequence of CsMYBF1 showed relative high similarity with the flavonol-related MYB proteins, and was most closely related to AtMYB111, with 89% identity in the R2R3 MYB domain, and 43% identity in the overall protein. The R2R3 MYB domain of CsMYBF1 was 84.3%, 81.5%, 88%, 80.6%, 84.3% identical to Arabidopsis AtMYB12, AtMYB11, grape VvMYBF1, maize ZmP1 and gentian GtMYBP3, respectively.

### Expression pattern and subcellular localization of CsMYBF1

To reveal the spatiotemporal expression of *CsMYBF1* in citrus, qRT-PCR analyses were performed using the total RNAs extracted from various organs of citrus and the pulp tissues at different development stages. As shown in Fig. 2b, transcript levels of *CsMYBF1* were high in the flower and young leaf but relative low in the mature leaf, seed and callus. In the pulp tissues, the mRNA level of *CsMYBF1* was the highest in 80 days after flowering (DAF), then decreased along with the fruit development and remained constant up to 170DAF. The expression features of *CsMYBF1* coincided with the accumulation profiles of citrus flavonoids and the transcript levels of the citrus flavonol-related structural genes^29^,^30^,^31^.

The subcellular distribution of CsMYBF1 were analysed using transient expression in a citrus protoplast system. OsGhd7-CFP was used as a positive control since OsGhd7 was localized in the nucleus^32^. The green fluorescence generated by CsMYBF1-GFP was distributed in the same area as that of the cyan fluorescence generated by OsGhd7-CFP, indicating that CsMYBF1 was a nuclear protein (Fig. 2c). Additionally, CsMYBF1-GFP was stably transformed into tomato, and root tip cells of positive transgenic tomato lines possessed strong fluorescence signals in the nucleus (Supplementary Figure S2). Both these results provided evidence to show that CsMYBF1 was a nucleus-localized protein and may function as a transcription factor.

### Effects of overexpression of CsMYBF1 on the metabolite and gene expression profiles in tomato fruits

**Figure 3:**
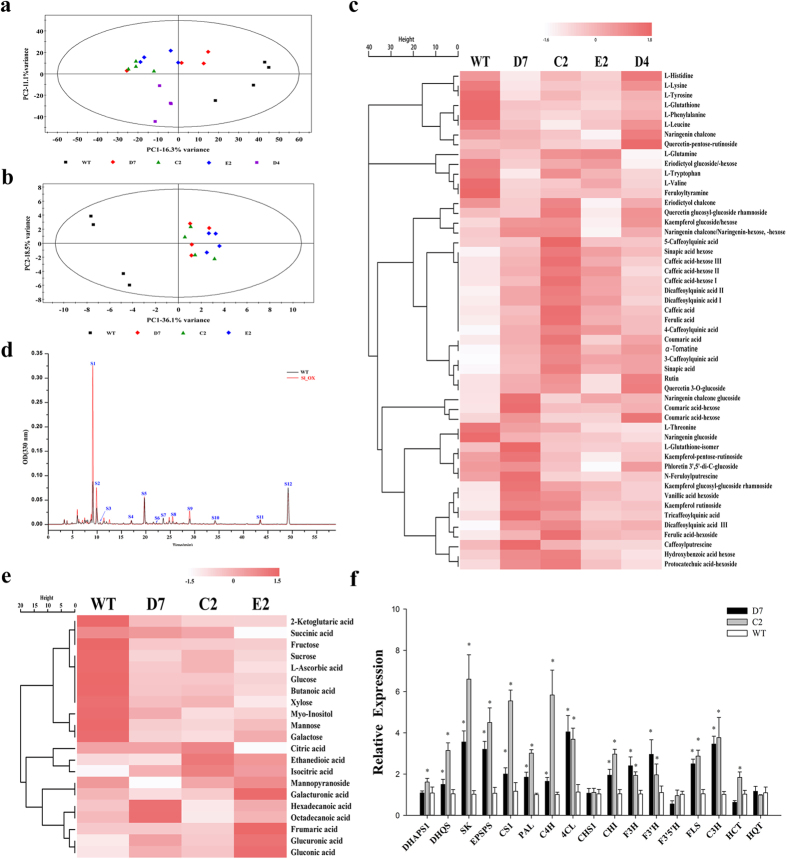
Integrated metabolome and transcriptome analysis for the *CsMYBF1*-overexpressing tomato fruits. Differences between metabolic profiles of WT/transgenic lines were adopted by PCA of LC-MS (**a**) and GC-MS data sets (**b**). (**c**) Hierarchical cluster analysis of secondary metabolites data in the WT and transgenic tomato fruits. (**d**) HPLC analysis of methanol extracts from WT and *CsMYBF1*-overexpressing tomato fruits. S1, 3-Caffeoylquinic acid; S2, 4-Caffeoylquinic acid; S3, Quercetin glucosyl-glucoside rhamnoside; S4, Kaempferol glucosyl-glucoside rhamnoside; S5, Rutin; S6, Phloretin 3′,5′-di-C-glucoside; S7, Kaempferol rutinoside; S8, Dicaffeoylquinic acid; S9, Dicaffeoylquinic acid; S10, Naringenin chalcone-glucoside; S11, Tricaffeoylquinic acid; S12, Naringenin chalcone (**e**) Hierarchical cluster analysis of primary metabolites data in the WT and transgenic tomato fruits. Data were processed with Z-score transformation and hierarchically clustered using Spearman distance. (**f**) Analysis of transcript levels of endogenous phenylpropanoid related genes in transgenic tomato fruits. Data are means ± SE of three replicate PCRs. Asterisks (P < 0.05, Student’s t-test) indicate significant differences compared with WT.

A total of 24 independent *CsMYBF1*-overexpressing tomato transformants were regenerated, with indistinguishable growth phenotype from that of the wild type (WT) plants. The chlorogenic acid contents in transgenic tomato flesh tissues detected by HPLC were associated with the transcript levels of *CsMYBF1* (Supplementary Figure S3). Together with the WT plants, progeny of four transgenic lines (lines D7, C2, E2 and D4) were selected for further analysis, according to their phenolic compound contents and high transcript levels of *CsMYBF1*. As indicated in Fig. 3a,b, principal component analysis (PCA) of both the LC-MS and GC-MS data sets could distinguish metabolite profiles of WT and transgenic lines.

Results of the metabolic profile found by LC-MS indicated that specific phenylpropanoid compounds were markedly altered by *CsMYBF1* expression (Table 1 and Fig. 3c). The caffeoyl quinic acids and dicaffeoyl quinic acids and the common hydroxycinnamic acid compounds in tomato, caffeic acid, ferulic acid and sinapic acid, except coumaric acid, were all significantly increased in all overexpression lines (Table 1). Additionally, their corresponding derivatives also showed enhanced levels in these transgenic lines. However, the detected flavonoids exhibited no significant differences between the WT and transgenic lines (Supplementary Table S2). The results were further confirmed by quantitative analyses of fruit methanol extracts using HPLC (Fig. 3d). The aromatic amino acid levels of phenylalanine, tryptophan and tyrosine were significantly reduced in some of the transgenic lines (Table 1).

GC-MS profiles revealed that the *CsMYBF1*-overexpressing tomato fruits displayed substantial changes in the level of primary metabolites (Fig. 3e and Supplementary Table S3). Sugars were the predominant compounds in total detected primary metabolites. Of the 6 sugars measured, fructose, glucose, mannose, galactose and sucrose, with the exception of xylose, all showed significant reduced levels in the overexpression tomato fruits. For the organic acids, the increased amount of isocitric acid and glucuronic acid and decreased levels of 2-ketoglutaric acid were observed in transgenic fruits.

A large number of differentially expressed genes in the overexpression lines were identified from the Digital Gene Expression (DGE) data (Supplementary Table S4). Nearly all the genes encoding enzymes of the shikimate pathway and the phenylpropanoid pathway were all significantly induced (Table 2). However, genes encoding chalcone synthase, the key rate-limiting enzyme in tomato flavonoid biosynthesis, were not observed in the differential genes list. Additionally, expression levels of large amount genes involved in primary metabolism were also significantly induced in transgenic lines. For example, a series of genes involved in glycolysis, pentose phosphate pathway and sucrose biosynthesis, like genes encoding enolase, glucose-6-phosphate 1-dehydrogenase and sucrose synthase, were expressed more highly in transgenic fruits than in WT fruits (Table 2). Expression levels of 17 selected phenylpropanoid related genes were further validated by quantitative RT-PCR and exhibited similar results (Fig. 3f). Taken together, ectopic expression of *CsMYBF1* in tomato significantly increased transcript levels of a series of genes involved in primary and secondary metabolism, reduced the contents of the major sugars and selectively increased the amount of hydroxycinnamic acid compounds. Inconsistent with expectations, the flavonol contents were not significantly altered in transgenic fruits, which could be due to the constant transcript levels of the key *CHS* genes.

### Effects of suppression of CsMYBF1 on the phenylpropanoid metabolism in citrus callus

To test the function of CsMYBF1 in a homologous system, RNA interference (RNAi) was used to suppress the expression of *CsMYBF1* in citrus callus cultures. We preliminary examined citrus callus cultures of different varieties, and confirmed that the ‘Guoqing’ NO. 1 satsuma mandarin (G1) callus line, accumulates large amount of phenolic acids and flavonol compounds and expresses a quantity of *CsMYBF1*. Therefore, the G1 callus line was selected for the RNAi experiments.

**Figure 4:**
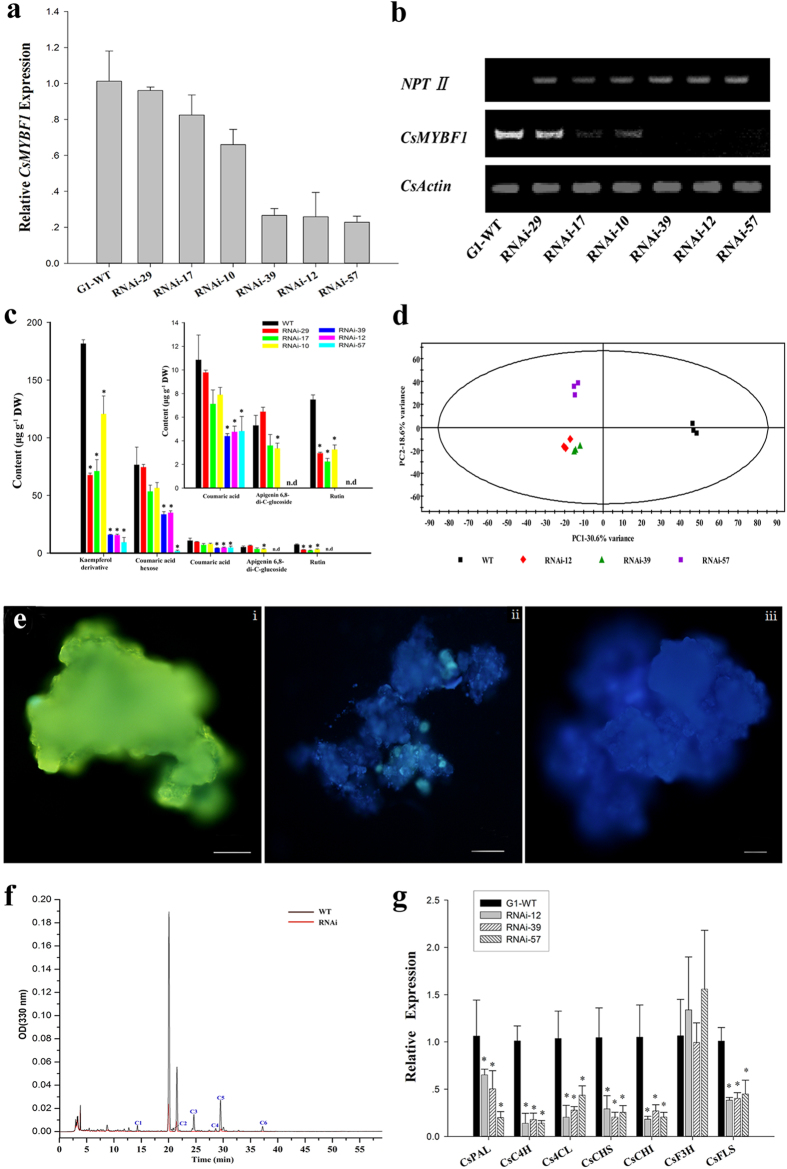
Effects of RNAi suppression of *CsMYBF1* in citrus callus on the endogenous gene expression and metabolite accumulation. Expression analyses of *CsMYBF1* expression in WT and transgenic lines by quantitative RT-PCR (**a**) and semi-quantitative RT-PCR (**b**). (**c**) Contents of five representative phenylpropanoid compounds in WT and transgenic callus lines determined by LC-MS. Data are means of three replicates and error bars indicating SD. DW, dry weight. Asterisks indicate significant differences as determined by t-test analysis (*P* < 0.05). (**d**) Differences between metabolic profiles of WT and RNAi callus lines detected by PCA of LC-MS data sets. (**e**) Visualized flavonol accumulation in transgenic calli. Representative images for the calli of WT (i), RNAi lines (ii) and negative control (iii) were shown. Bars = 1mm. (**f**) HPLC analysis of methanol extracts from WT and RNAi callus lines. C1, Kaempferol derivative; C2, Rutin; C3, Kaempferol-rutinside; C4, Quercetin derivative; C5, Kaempferol derivative; C6, Quercetin derivative (**g**) Analysis of transcript levels of endogenous phenylpropanoid biosynthesis genes in transgenic lines by quantitative RT-PCR. The WT expression data were normalized to 1. Data are means ± SD of two independent biological replicates. Asterisks (P < 0.05, Student’s t-test) indicate significant differences compared with WT.

Together with WT calli, six independent transformants with varying degrees of *CsMYBF1* transcript levels (Fig. 4a,b) were analyzed by LC-MS. The G1 callus accumulated various kinds of flavonol compounds, the coumaric acid and coumaric acid derivatives, aromatic amino acids, and flavone derivatives (Supplementary Table S5). The contents of the hydroxycinnamic acid and flavonoid compounds were reduced with varying degrees and exhibited correlations with *CsMYBF1* transcript levels in different callus lines (Fig. 4a–c). Representative results of five typical compounds (coumaric acid, coumaric acid hexose, apigenin 6, 8-di-C-glucoside, rutin and kaempferol derivative) were shown in Fig. 4c.

Three callus lines (RNAi-12, 39, 57) with greatest reduction in *CsMYBF1* expression and phenolic compound contents were further selected for PCA, indicating that the metabolic profiles of the WT and RNAi lines distinguished significantly (Fig. 4d). Flavonol accumulation in calli was visualized with diphenylboric acid 2-aminoethyl ester (DPBA), a flavonol-specific dye, which generates yellow-green fluorescence with kaempferol and yellow-gold with quercetin^33^. The yellow-green fluorescence was observed in the WT, indicating the mass accumulation of kaempferol compounds, which was consistent with the metabolite data identified by LC-MS. The *CsMYBF1* RNAi callus lines displayed only small amount of fluorescence spots, similar with that in negative control (callus does not accumulate detectable flavonols), indicating the sharp decrease of flavonol contents in the RNAi lines. Representative pictures were shown in Fig. 4e. The differences of the flavonol contents between genotypes were further validated by HPLC analysis (Fig. 4f).

Further qRT-PCR expression analysis revealed that the down-regulation of CsMYBF1 accompanied with proportionally reduced expression of most phenylpropanoid pathway genes: i.e. *CsPAL*, *CsC4H*, *Cs4CL*, *CsCHS*, *CsCHI* and *CsFLS* in the transgenic lines except *CsF3H* (Fig. 4g). Different from the unchanged transcript levels of *SlCHS* genes in transgenic tomato fruits, the expression levels of *CsCHS* (*CitCHS2*) were significantly reduced in the RNAi callus lines. These expression changes of the structural genes could clearly account for the alteration of the phenolic compound composition in RNAi lines. The above results indicated the essential roles of *CsMYBF1* in regulating the flavonol and hydroxycinnamic acid biosynthesis in citrus.

### CsMYBF1 activates promoters of the phenylpropanoid pathway genes in tomato and citrus

**Figure 5:**
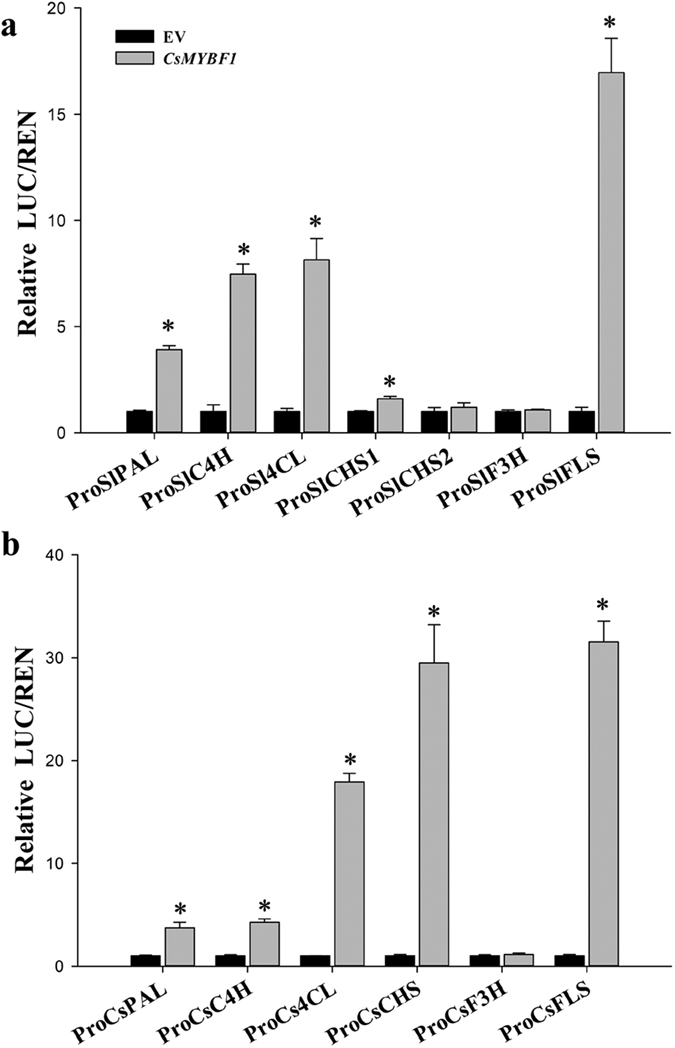
Effects of CsMYBF1 on promoter activities of phenylpropanoid pathway genes from tomato (**a**) and citrus (**b**) in transient expression assays. LUC, Firefly luciferase activity; REN, Renilla luciferase activity. The ratio of LUC/REN of the empty vector (EV) plus promoter was used as a calibrator (set as 1). Error bars indicate SE from six replicates. Asterisks indicate significant differences as determined by t-test analysis (*P* < 0.05).

To evaluate the ability of CsMYBF1 to transcriptionally activate phenylpropanoid pathway genes, tobacco leaves were coinfiltrated with *Agrobacterium* harboring the *CsMYBF1* overexpression construct together with a series of constructs containing a LUC reporter gene driven by the promoters of phenylpropanoid pathway genes. As illustrated in Fig. 5, CsMYBF1 activated the promoters of *SlPAL* (~approximately 4 fold compared with the control), *SlC4H* (~7 fold) and *Sl4CL* (~8 fold) in tomato. CsMYBF1 also induced the promoters of the *CsPAL* (~4 fold), *CsC4H* (~4 fold) and *Cs4CL* (~18 fold) in citrus, indicating its ability to activate the expression of the general phenylpropanoid pathway genes in both species. The promoters of the flavonol-specific *FLS* genes from two species were both greatly activated, whereas the activities of *F3H* promoters from two species were neither substantially altered by CsMYBF1. Interestingly, CsMYBF1 activated the *CsCHS* promoter approximately 30 fold, but the promoter activities of two *SlCHS* genes were not significantly activated. These results showed that CsMYBF1 activated the heterologous and homologous promoters of the general phenylpropanoid pathway genes and the flavonol-specific genes, but exhibited different specificity on the activities of *CHS* promoters from citrus and tomato.

### CsMYBF1 requires the MYBPLANT cis-elements for transactivation

**Figure 6:**
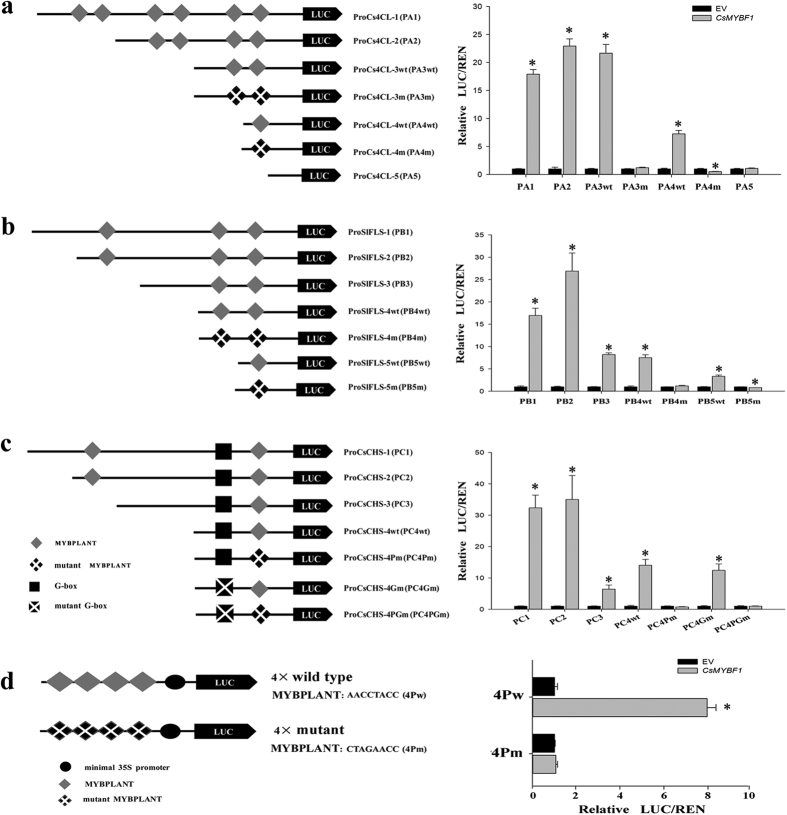
CsMYBF1 specifically activates the three selected promoters via the MYBPLANT cis-element. Diagrams showing various DNA fragments of the three selected promoters inked to the firefly luciferase reporter: (**a**) ProCs4CL, (**b**) ProSlFLS and (**c**) ProCsCHS. The detailed promoter lengths and the position of the cis-elements were shown in Figure S4. (**d**) Diagrams showing four copies of wild type/mutant MYBPLANT cis-elements linked to the minimal 35S promoter and the firefly luciferase reporter. The corresponding relative ratio of LUC/REN was shown on the right. The ratio of LUC/REN of the empty vector (EV) plus promoter was used as a calibrator (set as 1). Error bars indicate SE from six replicates. Asterisks indicate significant differences as determined by t-test analysis (*P* < 0.05).

To identify the motifs directly targeted by CsMYBF1, the *Cs4CL*, *SlFLS* and *CsCHS* promoters, which were strongly activated, were selected for further analysis. The MYBPLANT cis-element ([A/C] ACC [A/T]A[A/C]C) was regarded as a consensus MYB binding site for phenylpropanoid biosynthetic genes^34^. Sequence comparison revealed that cis-elements similar to MYBPLANT were presented in all these three selected promoters, indicating that these elements might be potential target motifs of CsMYBF1. As depicted in Fig. 6 (details in Supplementary Figure S4), a set of deletion and block mutation constructs were generated to test the functional roles of these motifs.

Six putative MYBPLANT cis-elements were identified in the promoter regions of *Cs4CL*, and deletions of the four elements in the distal region did not significantly affect the activation, whereas the deletions and mutations for the two elements in the proximal region greatly reduced the transactivation (Fig. 6a). Three putative MYBPLANT cis-elements located upstream of *SlFLS*. Transactivation of the *SlFLS* promoter by CsMYBF1 was reduced by each deletion of the MYBPLANT-like cis-elements. Comparably, deletions in regions without MYBPLANT-like elements did not cause significant reduction (Fig. 6b). The mutation assays results for the last two elements also supported the above observations. The transactivation assays for the two target elements in *CsCHS* promoter exhibited similar results (Fig. 6c). The G-box cis-element was characterized as an important motif in CHS promoter from Arabidopsis^34^. Mutations in G-box sequences were also introduced in truncated *CsCHS* promoter, and the results indicated that CsMYBF1 could activate *CsCHS* promoter via the MYBPLANT cis-element but not the G-box cis-element (Fig. 6c).

To further ascertain whether the MYBPLANT cis-elements could be specially activated by CsMYBF1, we constructed chimeric promoters containing four copies of wild type (in *CsCHS* promoter) or mutant MYBPLANT motifs followed by the minimal CaMV35S promoter (Pro35Smin), and fused these promoters to the LUC reporter. As shown in Fig. 6d, chimeric promoter containing wild type MYBPLANT motif was strongly activated by CsMYBF1, while mutation of the MYBPLANT motif completely abolished this activation. The above results indicated that the MYBPLANT cis-element was an essential motif for CsMYBF1 in activating the phenylpropanoid biosynthetic genes.

### CsMYBF1 specially binds to the MYBPLANT cis-elements

**Figure 7:**
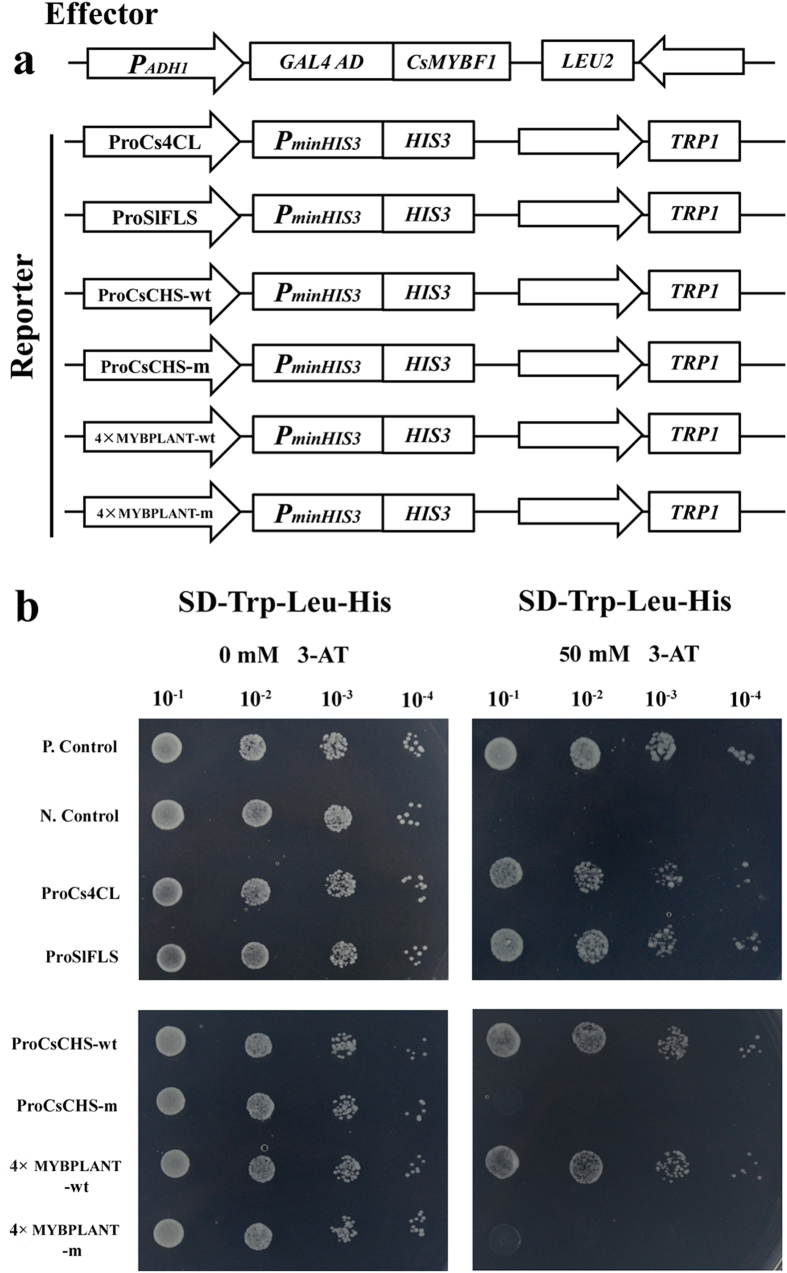
Yeast one-hybrid assays. (**a**) Schematic structures of the yeast one hybrid effector (pGADT7-CsMYBF1) and reporter vector. (**b**) Growth of yeast cells transformed with the effector plasmid and the reporter plasmid on SD-Trp-Leu-His supplemented with or without 50 mM 3-AT. P. Control, positive control (p53HIS2 plus pGAD-p53); N. Control, negative control (p53HIS2 plus pGAD-CsMYBF1)

To further confirm whether CsMYBF1 interacted with the target promoters directly, a yeast one-hybrid system was used. As shown in Fig. 7a,b, the interaction tests indicated that CsMYBF1 interacted directly with both promoters of *Cs4CL* and *SlFLS*. To test whether CsMYBF1 specially bound to the region of MYBPLANT motif in the *CsCHS* promoter, region of about 60 bp in length surrounding the MYBPLANT cis-element, and the same region with the mutant MYBPLANT sequences were used as baits for binding assays. It was evident that yeast co-transformed with the CsMYBF1 and the natural promoter fragment, but not with the CsMYBF1 and the corresponding mutant region, grew well in the selective medium. Additionally, quadruple repetitions of the wild type/mutant MYBPLANT fragments in the *CsCHS* promoter were separately cloned into the reporter construct for binding assays, and the interaction tests exhibited identical results (Fig. 7b).

**Figure 8:**
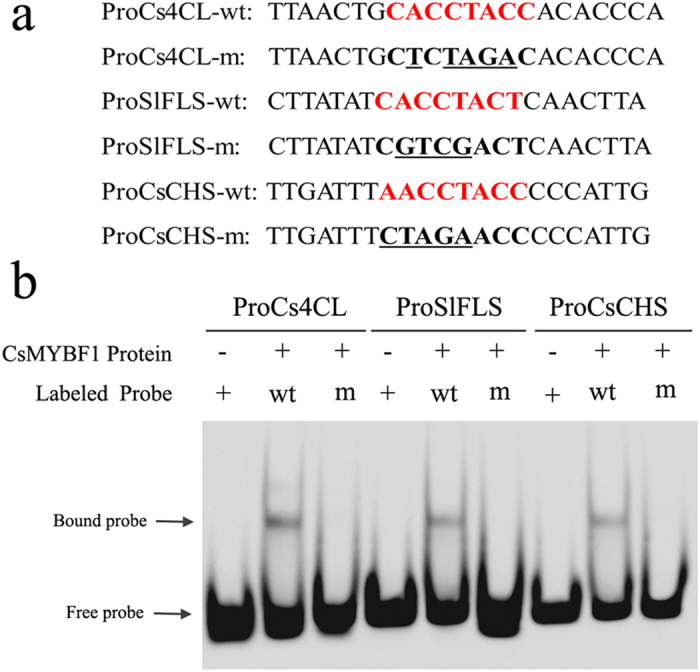
CsMYBF1 binds to the promoters containing MYBPLANT cis-elements. (**a**) Sequences for oligonucleotides used in the EMSA. The red bold letters highlight MYBPLANT sequences, mutated positions are underlined. (**b**) EMSA of the CsMYBF1 binding to the promoters of *Cs4CL*, *SlFLS* and *CsCHS*. The “+” and “−” indicate the presence and absence of the corresponding probe or protein, respectively. Arrows indicate the positions of protein-DNA probe complex and free probes, respectively.

We next performed electrophoretic mobility shift assays (EMSA) to further verify the physical interaction between CsMYBF1 protein and MYBPLANT cis-elements. The purified recombinant CsMYBF1 protein was incubated with Cy3-labeled promoter fragments of the three selected genes (Fig. 8a). As shown in Fig. 8b, a specific DNA complex was detected with all three wild type probes, which was not formed when the respective MYBPLANT cis-element was mutated. Above all, the results showed that CsMYBF1 can specifically bind to the promoters of *Cs4CL*, *SlFLS* and *CsCHS* through the MYBPLANT cis-elements.

## Discussion

In this study, a R2R3-MYB transcription factor gene, *CsMYBF1*, was isolated from citrus. The expression features of *CsMYBF1* coincided with the accumulation profiles of flavonoids in citrus, which were mainly biosynthesized in immature fruit and in young, rapidly growing leaves^29^,^30^. Accordingly, the mRNA levels of the flavonol-related structural genes were also high in these young active citrus tissues^31^. Additionally, the sugar contents in citrus pulp tissues were low in young fruits and increased along with the citrus fruit development^35^. The correlation between expression levels of *CsMYBF1* and the metabolites contents in citrus fruits may implicate the important role of *CsMYBF1* in the regulation of related metabolites.

The gene function was characterized through analysis of *CsMYBF1* overexpression lines in tomato and the RNAi lines in citrus callus. The functional role of CsMYBF1 that acted as the flavonol regulator was validated in the RNAi lines. In transgenic tomato fruits, the general phenylpropanoid pathway genes were coordinately unregulated and the contents of the hydroxycinnamic acids were significantly induced. On the contrary, the hydroxycinnamic acid and its derivatives were significantly reduced in the RNAi lines. The opposite metabolite changes between the overexpression and RNAi lines provided strong evidence to support the function of *CsMYBF1* in regulating the hydroxycinnamic acid biosynthesis. It was speculated that the regulation of hydroxycinnamic acid biosynthesis may be an additional and perhaps ancient function of the flavonol-related MYB proteins^12^.

It was thought that the flavonol-related MYB proteins have a preference for early biosynthesis genes in the flavonoid pathways as their target genes^36^. In this study, the *CsCHS* and *CsFLS* genes were both significantly induced by CsMYBF1. It was notable that the *CsF3H* gene, which belongs to early flavonoid biosynthetic genes in citrus, was not activated by CsMYBF1 in transactivation assays. The results could account for the unaltered expression level of *CsF3H* in the transgenic callus lines. *F3H* was responsible for the production of dihydroflavonol, the intermediate for the flavonol biosynthesis. *In vitro* experiments revealed that the citrus FLS enzyme harboured an intrinsic F3H-like activity as well^37^. Therefore we speculated that the regulation of the flavonol biosynthesis by CsMYBF1 may not involve *CsF3H*. In addition to flavonols, *CsMYBF1* was also involved in controlling the accumulation of flavones derivatives in callus, suggesting a wide role of this R2R3-MYB regulator in the control of phenylpropanoid compounds. Above all, *CsMYBF1* was an essential transcription factor for the regulation of the phenylpropanoid biosynthesis in citrus, and could serve as effective molecular tool for the breeding of citrus with optimized phenylpropanoid levels and compositions.

The constitutive overexpression of *CsMYBF1* resulted in significant metabolite alterations in tomato fruits, including the elevated levels of a subset of hydroxycinnamic acid derivatives and reduced amounts of major sugars. The transcriptome data indicated that the expression of high number of genes in primary and secondary metabolism were significantly affected, such as genes involved in glycolysis, the pentose phosphate pathway and the phenylpropanoid pathway. Elevated expression of the genes involved in primary metabolism could increase the supply of precursors for secondary metabolism^38^. A series of significantly induced genes in primary metabolism implicated that there may be greater carbon flux through glycolysis and the pentose phosphate pathway to drive the biosynthesis of secondary metabolic pathways in *CsMYBF1* overexpression fruits. As a result, the contents of the major sugars were significantly reduced, and the enhanced carbon flux could contribute to the increasement of phenylpropanoid contents in transgenic fruits.

In this study, overexpression of *CsMYBF1* not only activate the genes of the phenylpropanoid pathway, but also reprogram the primary metabolism and change the carbon flux in tomato fruits. The homologous transcription factor of CsMYBF1 in *Arabidopsis*, AtMYB12, has also been reported to exhibit similar metabolic effects when overexpressed in tomato fruits, which can activate genes encoding enzyme of both primary metabolism and the phenylpropanoid metabolism, and control both the supply and demand of the carbon^38^. Similarly, homologous gene to *CsMYBF1* in maize, *P1*, could also modify the expression of high number of genes including those involved in glycolysis^18^. So we speculate that the flavonol-related MYB transcription factors *CsMYBF1* could also affect the genes involved in primary metabolism simultaneously, to ensure adequate supply of precursors for the phenylpropanoid biosynthesis.

Interestingly, the contents of flavonols and even all the flavonoids compounds in tomato fruits were not altered when overexpressing this flavonol regulator. This was beyond our expectations and quite different from other known flavonol-related MYB proteins when overexpressed in tomato^12^,^14^. Transcriptome data revealed that the *CHS* genes, encoding the rate-limiting enzyme of flavonoid biosynthesis, were not significantly induced in transgenic tomato, which may explain the constant flavonoid contents in *CsMYBF1*-overexpressing tomato fruits.

CHS play a pivotal role in flavonoid biosynthesis and the loss of CHS activity exerts significant metabolic effects in many plant species^8^. In *CHS* RNAi tomato plants, the contents of key flavonoids such as naringenin chalcone and rutin were dramatically reduced^39^. In Arabidopsis, the *tt4* mutant plants, defective in CHS, showed the absence of downstream flavonols^40^. Ectopic concomitant expression of *CHS* and *FLS* induced the accumulation of flavonols in tomato flesh tissues, while either gene alone was insufficient. CHS and FLS have been regarded as key biosynthetic enzymes that determined accumulation of flavonols throughout the tomato fruit tissue^41^. The transcript levels of *CHS* genes were all significantly increased in transgenic tomato fruits, where induced flavonol accumulation was observed^12^,^14^,^42^. In our study, the expression of *CHS* genes was not altered in the transgenic tomato, albeit a set of other structural genes in flavonoid pathway were all significantly induced. Therefore, the CHS activity became the main limiting factor for flavonol accumulation in *CsMYBF1*-overexpressing tomato fruits.

The promoter activation assays indicated that CsMYBF1 significantly activated both promoters of *FLS* genes from tomato and citrus, consisted with the role of the flavonol regulator. However, CsMYBF1 only activated the promoter of *CsCHS* but not of *SlCHS*, which could lead to different effects on the alterations of phenylpropanoid metabolites. Such differential activation of *CHS* promoters by regulatory factors were also observed in previous studies. The combination of the anthocyanin-related regulators, C1 and R or AN2 and JAF13, can activate the promoter of *CHS* from maize, but not from petunia. The failure to active the petunia *CHS* promoter led to the anthocyanin pigmentation pattern that was not fundamentally altered in petunia plants when co-expressing AN2 and JAF13^43^. Similarly, the failure to activate the tomato *CHS* promoters could give rise to the constant flavonoid levels in *CsMYBF1*-overexpression tomato fruits. Some potential factors that affect the transactivation of *CHS* promoters may be responsible for such regulatory differences.

The *CHS* promoters have been well studied in many other plant species. The MRE was widely distributed in *CHS* promoters from different species and required for the activation by many flavonol-related MYB proteins^11^,^44^. MYBPLANT motif is a common MYB-binding cis-acting element and frequently resides in promoters of the genes involved in phenylpropanoid biosynthesis^34^. The MRE sequence 5′-ACCTACC-3′ was identical with most regions of the conserved MYBPLANT cis-elements, and these two motifs were actually related to the same sequences in many cases. A series of deletions and mutation analyses indicated that CsMYBF1 could activate a set of target promoters through MYBPLANT cis-element, but not other motifs like G-box cis-element. Therefore, the MYBPLANT cis-element could also act to confer the *CHS* promoter activation in response to CsMYBF1. The cis-elements play an important role in determining the target gene specificity of MYB proteins, changes in the cis-elements sequences may affect the biochemical interactions with transcription factors and cause the phenotypic differences^36^,^45^. Therefore, the failure of CsMYBF1 to activate *SlCHS* promoters may be due to the corresponding elements in tomato have already diverged away to be recognized by CsMYBF1.

However, the distribution of the target motifs alone cannot fully explain the observed target gene specificity of CsMYBF1. Other factors like the surrounding sequences of the cis-elements and the numbers of cis-elements, may also affect the transactivation. For example, the *CsF3H* was not significantly activated by CsMYBF1, albeit the conserved MYBPLANT motif was identified in its promoter region. Additionally, differences in protein sequences of CsMYBF1 to closely related MYB genes of other species, especially variation in particular amino acid domains, also possibly leading to different specificity to CHS promoters.

Taken together, we speculated that the flavonol regulator CsMYBF1 specially increased the hydroxycinnamic acid contents and was not involved in the regulation of flavonol biosynthesis could be due to the failure to activate *CHS* promoters in the heterologous expression system. Therefore, *CsMYBF1* could be considered as a valuable candidate gene for plant metabolic engineering, which can significantly affect primary metabolism and increase flux to phenylpropanoid metabolites of interest without incurring effects on the flavonoid contents.

## Methods

### Isolation of CsMYBF1 cDNA and phylogenetic analysis

The full-length of the *CsMYBF1* cDNA was obtained using the 5′RACE kit and 3′RACE kit (Takara) following the supplier’s protocol. The primers were listed in Supplementary Table S6. Different protein sequences were aligned and edited using the Clustal W program and GeneDoc software. A neighbor-joining tree was built up using the MEGA 5.0 software with the bootstrap values from 1000 replicates.

### Plant materials

Samples of leaves (young and mature stages), callus, flowers, seeds and pulp from different fruit development stages were all derived from the ‘Anliu’ sweet orange (*Citrus sinensis* L. Osbeck) in Institute of Citrus Research of Guangxi. All samples were frozen in liquid nitrogen immediately, and stored at −80 °C until use. The G1 callus was maintained on solid MT basal medium and subcultured every 20 days. The transgenic tomato plants were grown under standard glasshouse conditions. The *Nicotiana benthamiana* was planted in a growth chamber controlled at 14 h light, 10 h dark, and 24 °C cycles.

### Subcellular protein localization

The 35S:CsMYBF1-GFP fusion construct was produced by inserting the full ORF of *CsMYBF1* into the pM999-35 vector. Plasmids were extracted and purified using the Plasmid Midi Kit (QIAGEN) following manufacturer’s protocol. The isolation of the mesophyll protoplast of HB pummelo (*Citrus grandis* (L.) Osbeck) was carried out according to Grosser and Gmitter^46^. The OsGhd7 in rice was used as a nuclear maker. The 35S:CsMYBF1-GFP and 35S:OsGhd7-CFP plasmids were co-transformed into citrus protoplasts according to the procedures described by Fang *et al*.^32^. The root tip cells of the positive stable overexpression tomato lines for 35S:CsMYBF1-GFP were also used. The florescence images were captured by using a confocal laser-scanning microscope (TCS SP2, Leica, Germany).

### Generation of constructs and transformation

The full length of *CsMYBF1* was ligated into binary vector PBI121 driven by 35S promoter. The fragment of *CsMYBF1* ORF (428 bp length, 562-989 bp) was recombined into the gene silencing vector pHGRV by BP reaction. These constructs were electroporated into *Agrobacterium* strain EHA105. The overexpression and RNAi vector was transformed into tomato and callus, respectively.

Tomato variety Ailsa Craig (AC) was transformed according to the method of Fillatti *et al*.^47^. The transformation for callus line was performed according to Duan *et al*.^48^. The putative transgenic lines were examined by semi-quantitative RT-PCR using target gene specific primer (GSP-CS) and the NPTII gene-specific primer (NPTII) (Supplementary Table S6).

### RNA isolation and quantitative analysis of gene expression

Total RNA was isolated using a modified Trizol extraction protocol^49^. First-strand cDNA was synthesized using PrimScript™ RT reagent Kit with gDNA Eraser (Takara). Quantitative real-time RT-PCR was performed on ABI Prism 7900HT (Applied Biosystems) with the SYBR Green system. The gene specific primers used in qRT-PCR analysis were listed in Supplementary Table S6. The *Actin* gene was used as internal control.

### Transcriptome analysis

Two independent transgenic tomato plants (line D7 and C2) and the WT plants were divided into two groups (overexpression/WT) and each with two independent biological replicates. Fruit samples from at least five plants of each genotype, which were harvested between 7 and 10 days after breaker stage, were pooled for total RNA isolation. The DGE experiments were performed by Novogene Bioinformatics Technology Co. Ltd (Beijing). Total reads were mapped to the tomato genome (http://solgenomics.net/) using Top Hat (2.0.9) software^50^. Differential expressed genes between the transgenic and WT plants were defined with the edgeR^51^, based on log_2_ fold change >1 and adjusted P value < 0.05. DGE results have been submitted to NCBI with accession number GSE73300.

### Secondary metabolite analysis

Secondary metabolite analysis for the methanol extract of the freeze-dried sample was performed on an Agilent 1200 series Rapid Resolution HPLC system hyphenated with an Agilent 6520 QTOF-MS/MS system (Agilent Technologies, Palo Alto, CA, USA). The HPLC separation was carried out on a Zorbx Eclipse Plus C18 1.8 μm, 2.1 × 100 mm reverse-phase analytical column operated at 35 °C. The mobile phase, which consisted of a 0.1% formic acid in Milli-Q water (A) and 0.1% formic acid in acetonitrile (B), was delivered at a flow rate of 0.3 mL min^−1^ under a gradient program: 0 min −5% B; 20 min −95% B; 22 min −95% B; 22.1 min −5% B; 28 min −5% B.

Putative metabolite identification was achieved by searching the accurate m/z values of the peaks against the tomato fruit specific databases Komics (http://webs2.kazusa.or.jp/komics) and the public databases (http://www.massbank.jp, https://metlin.scripps.edu), with a maximum deviation of observed mass to calculated mass of 5 ppm. For some other compounds, the identification was based on the information from published literatures. Candidate metabolites were further confirmed according to the corresponding in-source fragments and the retention times, if present, and using commercially available authentic standards. The putative compounds were quantified by calculating the area of each individual peak and comparing this to calibration curves obtained from the corresponding closest authentic standards. HPLC analysis was conducted according to the methods described in Chen *et al*.^52^ with some modifications.

### Flavonol staining of calli

The flavonol accumulation in different calli was visualized by diphenylboric acid 2-aminoethyl ester (DPBA), as described by Stracke *et al*.^53^. The calli were stained to saturation for at least 0.5 h in the solution of 0.25% (w/v) DPBA (Sigma D9754-1 G), 0.005% (v/v) TritonX-100. Fluorescence images were visualized under UV light on a universal fluorescence microscope (Olympus BX61, Tokyo, Japan).

### GC-MS analysis

The GC-MS analysis of the methanol extract of the fresh sample from the overexpression/WT tomato fruit was carried out by using a Thermo Trace GC Ultra coupled with DSQ II mass spectrometer (Thermo Fisher), based on the methods described in Yun *et al*.^54^. The processed data were used for PCA using the SIMCA- P 11.5 program (Umetrics).

### Dual luciferase assay of transiently transformed tobacco leaves

The putative promoters (approximately 1 kb upstream of the ATG start codon) of a series of phenylpropanoid biosynthesis genes were amplified in citrus and tomato respectively; the oligonucleotide sequences containing four tandem repeats of wild type/mutant MYBPLANT cis-elements were directly synthesized; various deletions of the promoter fragments were PCR-amplified from tomato and citrus genomic DNA; the block mutations for the MYBPLAT cis-elements in the corresponding promoter sequences were directly synthesized or amplified by recombinant PCR, all these fragments were inserted into the cloning site of pGreen0800-LUC. The full length of *CsMYBF1* mRNA was amplified and cloned into the effector vector (Modified PCAMBIA1380) driven by 35 S promoter. All the primers used were listed in Supplementary Table S6. Agrobacterium infiltration and measurement of the luciferase activities were conducted as described previously^55^,^56^.

### Yeast one-hybrid assays

The oligonucleotide sequences containing four tandem repeats of wild type/mutant MYBPLANT cis-elements, and other corresponding promoter sequences were cloned into the pHIS2 to generate the reporter vector. The full-length ORF of *CsMYBF1* was amplified by PCR and fused to the GAL4 activation domain in the vector pGADT7-Rec2 (Clonetech) to create a fusion protein pGADT7-CsMYBF1 (effector vector). All of the constructs were co-transformed into yeast strain Y187 according to the manufacturer’s instructions. The positive clones were subsequently incubated in SD (-Leu, -Trp) liquid media to an OD_600_ of 0.1(10^−1^) and diluted in a 10 × dilution series. From each dilution, 5 μL was spotted on SD (-His, -Leu, -Trp) media plates supplemented with 0 or 50 mM 3AT (Sigma-Aldrich).

### EMSA

The full length of *CsMYBF1* was ligated into pET28a vector, and the resultant pET28a-CsMYBF1 vector was introduced into *Escherichia coli* BL21(DE3). The His-tagged protein was purified with the nickel-nitrilotriacetic acid (Ni-NTA) agarose column. The Cy3-labeled oligonucleotide probes (in Fig. 8a) were directly synthesized and annealed. Binding reactions were performed using a solution containing 0.1% Nonidet P-40, 1 mM benzamidine, 0.5 mM phenylmethylsulfonyl fluoride (PMSF), 0.5 mM DTT, 50 μg/mL BSA and 100 ng/μL poly (dI-dC). A total of 5 μM Cy3-labeled probes and 1 μg purified His-CsMYBF1 protein were added to final volume of 20 μL and incubated at 4 °C for 2 h. The resulting DNA-protein complexes were loaded onto a pre-run 6% polyacrylamide gel. Electrophoresis were performed at 4 °C using 0.5×TBE (Tris-Borate-EDTA) as the electrophoresis buffer. Gel images were acquired using Amersham^TM^ Imager 600 (GE Healthcare).

## Additional Information

**How to cite this article**: Liu, C. *et al*. Characterization of a Citrus R2R3-MYB Transcription Factor that Regulates the Flavonol and Hydroxycinnamic Acid Biosynthesis. *Sci. Rep*. **6**, 25352; doi: 10.1038/srep25352 (2016).

## Supplementary Material

Supplementary Information

Supplementary Table S1

Supplementary Table S2

Supplementary Table S3

Supplementary Table S4

Supplementary Table S5

Supplementary Table S6

## Figures and Tables

**Figure 1:**
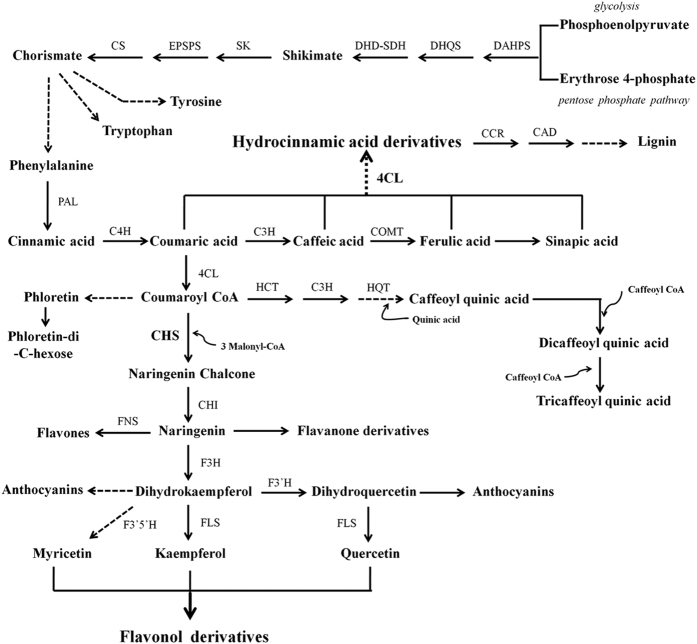
Schematic representation of shikimate and phenylpropanoid biosynthesis pathways in plants. Enzyme names are abbreviated as follows: DHAPS, DAHP synthase; DHQS, dehydroquinate synthase; DHD-SDH, 3-dehydroquinate dehydratase/shikimate 5-dehydrogenase; SK, shikimate kinase; EPSPS, EPSP synthase; CS, chorismate synthase; PAL, phenylalanine ammonia lyase; C4H, cinnamate 4-hydroxylase; 4CL, 4-hydroxy-cinnamoyl CoA ligase; C3H, ρ-Coumarate 3-hydrolase; COMT, caffeic acid 3-O-methyltransferase; CCR, cinnamoyl CoA reductase; CAD, cinnamyl alcohol dehydrogenase; CHS, chalcone synthase; CHI, chalcone isomerase; F3H, flavanone-3-hydroxylase; F3′H, flavonoid-3′-hydroxylase; F3′5′H, flavonoid-3′5′-hydroxylase; FLS, flavonol synthase; FNS, flavone synthase; HCT, cinnamoyl CoA shikimate/quinate transferase; HQT, hydroxycinnamoyl CoA quinate transferase.

**Table 1:** Changes in secondary metabolites in *CsMYBF1*-overexpressing tomato fruits relative to the WT.

Trend	Putative metabolite name	Content (mg/100 g DW)				
		WT	D7	C2	E2	D4
Up	Caffeic acid	1.67 ± 0.45	**4.32 ± 1.27**	**9.74 ± 4.51**	**5.33 ± 0.68**	**3.96 ± 0.12**
Up	Ferulic acid	1.1 ± 0.51	**2.68 ± 0.65**	**4.85 ± 2.19**	**2.82 ± 0.24**	**2.51 ± 0.65**
Up	Sinapic acid	0.75 ± 0.25	**1.89 ± 0.78**	**3.1 ± 1.05**	**2.12 ± 0.59**	**2.41 ± 0.41**
Up	3-Caffeoylquinic acid	24.56 ± 8.12	**85.83 ± 29.13**	**132.8 ± 41.71**	**105.86 ± 6.52**	**108.37 ± 13.55**
Up	4-Caffeoylquinic acid	2 ± 0.68	**5.13 ± 2.29**	**7.02 ± 0.54**	**6.11 ± 0.31**	**4.55 ± 0.6**
Up	5-Caffeoylquinic acid	1.25 ± 0.35	**2.86 ± 0.4**	**4.35 ± 0.39**	**3.26 ± 0.39**	**2.99 ± 0.69**
Up	Caffeic acid-hexose I	46.64 ± 21.16	81.98 ± 25.5	**148.84 ± 6.93**	**103.18 ± 19.37**	66.4 ± 18.08
Up	Caffeic acid-hexose II	8.88 ± 1.99	14.87 ± 5.44	**20.87 ± 2.57**	**17.73 ± 3.72**	10.13 ± 2.55
Up	Caffeic acid-hexose III	34.8 ± 12.79	**90.07 ± 21.14**	**171.25 ± 67.81**	**100.2 ± 15.18**	**62.24 ± 5.48**
Up	Ferulic acid-hexose	11.96 ± 2.68	**23.76 ± 4.75**	**39.49 ± 14.82**	**23.3 ± 3.47**	**21.18 ± 4.45**
Up	Sinapic acid hexose	1.51 ± 0.26	**5.6 ± 1.93**	**10.29 ± 3.56**	**7.54 ± 2.35**	**5.52 ± 2.11**
Up	Dicaffeoylquinic acid I	3.39 ± 1.68	13.98 ± 11.58	**20.1 ± 2.23**	**15.66 ± 1.77**	6.95 ± 3.15
Up	Dicaffeoylquinic acid II	2.8 ± 1.76	14.09 ± 10.57	**20.48 ± 5.99**	**14.16 ± 0.68**	**8.53 ± 1.7**
Up	Dicaffeoylquinic acid III	14.51 ± 6.25	67.66 ± 44.24	**75.64 ± 25.16**	**47.85 ± 5.52**	**53.8 ± 7.82**
Up	Vanillic acid hexoside	1.04 ± 0.65	3.04 ± 1.98	**7.06 ± 2.9**	**6.5 ± 2.17**	**3.33 ± 1.74**
Up	Protocatechuic acid-hexoside	1.27 ± 0.63	7.03 ± 6.4	**8.32 ± 3.82**	**3.12 ± 1.33**	1.06 ± 0.34
Down	L-Tyrosine	22.02 ± 6.53	**7.6 ± 2.49**	12.62 ± 4.92	**9.4 ± 0.96**	13.14 ± 5.96
Down	L-Phenylalanine	53.17 ± 4.01	**23.81 ± 11.53**	**19.8 ± 7.53**	**23.64 ± 3.58**	**31.01 ± 11.42**
Down	L-Tryptophan	19.18 ± 2.7	**9.18 ± 5.51**	17.91 ± 4.15	**14.18 ± 1.37**	**10.97 ± 4.46**
No change	Coumaric acid	3.32 ± 2.2	6.35 ± 3.27	**9.45 ± 3.82**	4.6 ± 0.57	6.92 ± 2.65
No change	Rutin	34.76 ± 11.31	47.71 ± 14.64	55.34 ± 13.6	35.08 ± 9.25	**60.4 ± 5.31**
No change	Kaempferol rutinoside	0.66 ± 0.28	0.56 ± 0.12	0.76 ± 0.36	0.7 ± 0.29	0.47 ± 0.1
No change	Eriodictyol chalcone	2.13 ± 0.93	1.71 ± 0.79	2.24 ± 0.54	1.4 ± 0.13	1.94 ± 0.29
No change	Naringenin chalcone	111.97 ± 59.58	96.91 ± 26.25	84.6 ± 26.53	55.85 ± 8.89	131.43 ± 30.63

DW, dry weight. Data presenting mean value ± SD of four biological replicates. Values in bold denote significant differences as determined by *t*-test analysis (*P* < 0.05). The compounds contents differ significantly from WT in at least two out of four transgenic lines as indicated in the status row: Up, upregulated; Down, downregulated; while differ significantly only in one transgenic line or no significant alterations compared to WT were labeled ‘No change’ (some representative compounds were listed).

**Table 2:** Gene expression changes for selected genes which were significantly induced in *CsMYBF1*-overexpressing tomato fruits.

Gene_id	Log_2_ Fold Change	*P* adj.	Description
Glycolysis			
Solyc03g093520.2	1.67	0.0367	6-phosphofructokinase
Solyc12g095880.1	1.88	0.0048	6-phosphofructokinase
Solyc04g072800.2	2.46	0.0012	Phosphoglycerate mutase
Solyc10g085550.1	2.17	0.042	Enolase
Solyc03g114500.2	3.05	1.96E-05	Enolase
Solyc06g076650.2	1.63	0.0212	Enolase
Solyc09g008840.2	1.33	0.0466	Pyruvate kinase
Solyc06g051930.2	1.4	0.0466	Pyruvate kinase
Solyc11g045370.1	1.91	0.0443	Lactate dehydrogenase
Solyc04g064710.2	6.75	0.0486	Alcohol dehydrogenase
Solyc06g072160.2	3.62	0.0001	Alcohol dehydrogenase
Pentose phosphate pathway			
Solyc05g015950.2	1.78	0.0101	Glucose-6-phosphate 1-dehydrogenase
Solyc02g093830.2	2.29	0.0002	Glucose-6-phosphate 1-dehydrogenase
Solyc12g014380.1	1.89	0.0026	Glucose-6-phosphate isomerase
Sucrose biosynthesis			
Solyc07g042550.2	2.15	0.0005	Sucrose synthase 1
Solyc03g098290.2	2.74	0.0002	Sucrose synthase
Solyc02g081300.2	2.25	0.0366	Sucrose synthase
Solyc07g007790.2	1.48	0.0178	Sucrose phosphate synthase
Solyc10g081660.1	1.67	0.0159	Sucrose phosphatase
Shikimate pathway			
Solyc02g083590.2	1.43	0.0353	Dehydroquinate synthase
Solyc01g067750.2	2.17	0.001	3-dehydroquinate dehydratase/shikimate dehydrogenase
Solyc04g051860.2	2.4	0.0004	Shikimate kinase
Solyc05g050980.2	2.87	0.0012	EPSP synthase
Solyc04g049350.2	2.68	4.43E-05	Chorismate synthase 1
Solyc02g088460.2	1.9	0.0115	Chorismate mutase
Phenylpropanoid pathway			
Solyc00g282510.1	3.31	0.0019	Phe ammonia-lyase
Solyc09g007900.2	2.65	0.0011	Phe ammonia-lyase
Solyc09g007910.2	3.5	2.08E-07	Phe ammonia-lyase
Solyc09g007920.2	3.82	4.44E-08	Phe ammonia-lyase
Solyc10g011930.1	4.38	0.0048	Phe ammonia-lyase
Solyc10g086180.1	3.82	1.45E-05	Phe ammonia-lyase
Solyc09g007930.2	1.95	0.0172	Phe ammonia-lyase
Solyc05g047530.2	3.81	0.0006	Cinnamate 4-hydroxylase
Solyc03g117870.2	2.01	0.0027	4-Coumarate-CoA ligase
Solyc03g097030.2	2.58	0.0037	4-Coumarate-CoA ligase
Solyc10g078240.1	2.17	0.0039	Coumarate 3-hydrolase
Solyc08g076780.1	4.52	0.001	Cinnamoyl-CoA reductase
Solyc05g052240.2	1.6	0.0201	Chalcone isomerase
Solyc02g083860.2	1.45	0.0295	Flavanone 3-hydroxylase
Solyc03g115220.2	2.38	0.0031	Flavonoid 3′-hydroxylase
Solyc11g013110.1	1.95	0.0026	Flavonol synthase
Solyc02g089770.2	1.91	0.0036	Dihydroflavonol 4-reductase
Solyc08g006770.2	2.53	0.0006	Anthocyanidin synthase
