# Supplementary material for: Characterization of a Citrus R2R3-MYB Transcription Factor that Regulates the Flavonol and Hydroxycinnamic Acid Biosynthesis
Source: Sci Rep. 2016 May 10;6:25352. doi: 10.1038/srep25352 (PMC4861916; doi:10.1038/srep25352)
Supplement: Supplementary Information [file srep25352-s1.doc]

**Supplementary Information**

**Characterization of a Citrus R2R3-MYB Transcription Factor That Regulates the Flavonol and Hydroxycinnamic Acid Biosynthesis**

**Chaoyang Liu^1^, Jianmei Long^1^, Kaijie Zhu^1^, Linlin Liu^1^, Wei Yang^1^, Hongyan Zhang^1^, Li Li^2^, Qiang Xu^1^,**

**Xiuxin Deng^1^,***

**^1^**Key Laboratory of Horticultural Plant Biology (Ministry of Education), Huazhong Agricultural University, Wuhan 430070, P.R.China.

**^2^** Robert W. Holley Center for Agriculture and Health, USDA-ARS, Plant Breeding and Genetics Section, School of Integrative Plant Science, Cornell University, Ithaca, NY 14853, USA

******Corresponding author*

**Corresponding author:**

Xiuxin Deng

Professor

Key Laboratory of Horticultural Plant Biology (Ministry of Education), Huazhong Agricultural University, Wuhan 430070, P.R.China

Tel number: +86-27-87286906

Fax number: +86-27-87384670

Email: [xxdeng@mail.hzau.edu.cn](mailto:xxdeng@mail.hzau.edu.cn)

**Supplementary Information**

**Supplementary Figures**

**Supplementary Figure S1** Protein alignment of R2R3-MYB-type transcriptional regulators of flavonol synthesis from citrus, *Arabidopsis*, grape and Japanese gentian and presumptive flavonol regulators from other plant species.

**Supplementary Figure S2** The subcellular localization of CsMYBF1 protein in the transgenic tomato root tip cells.

**Supplementary Figure S3** Chlorogenic acid contents (a) and transcript levels of *CsMYBF1* (b) in flesh tissues of the wild type and T_0_ transgenic tomato fruits.

**Supplementary Figure S4** Detailed schematic diagram of various DNA fragments of the three selected promoters inked to the firefly luciferase reporter. Promoter sequences of *Cs4CL* (a), *SlFLS* (b), *CsCHS* (c).

**Supplementary Tables**

**Supplementary Table S1** Accession numbers of the MYB transcription factors in the phylogenetic tree.

**Supplementary Table S2** Tentatively identified metabolites in the LC-MS analysis and their abundancy in tomato fruits.

**Supplementary Table S3** Changes in primary metabolites in *CsMYBF1*-overexpressing tomato fruits relative to the WT.

**Supplementary Table S4** Global digital gene expression profiling data.

**Supplementary Table S5** Tentatively identified metabolites in the LC-MS analysis and their abundancy in citrus callus.

**Supplementary Table S6** Primers used in this study.


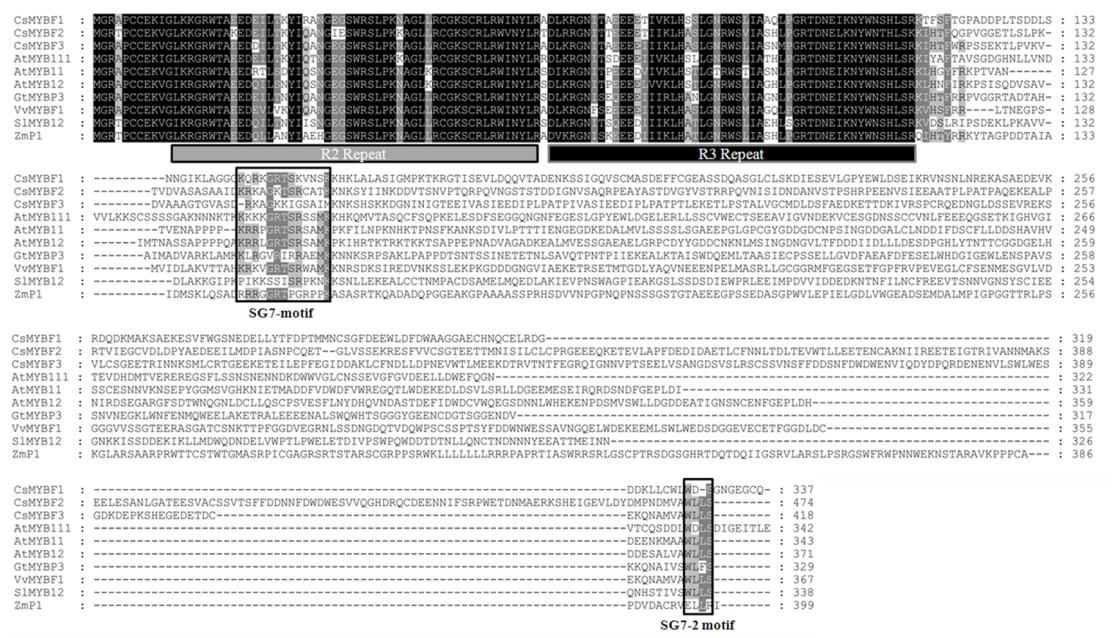


**Supplementary Figure S1** Protein alignment of R2R3-MYB-type transcriptional regulators of flavonol synthesis from citrus, *Arabidopsis*, grape and Japanese gentian and presumptive flavonol regulators from other plant species.


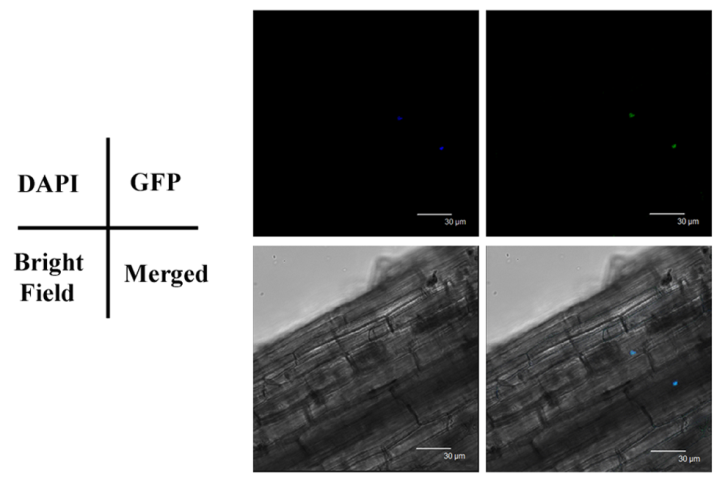


**Supplementary Figure S2** The subcellular localization of CsMYBF1 protein in the transgenic tomato root tip cells.


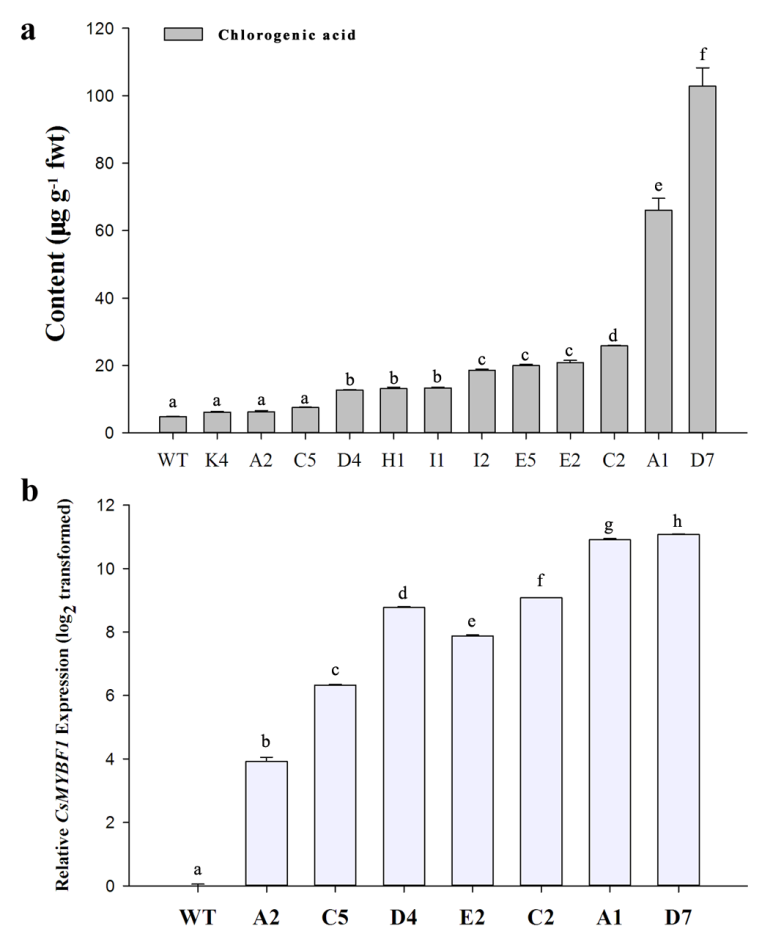


**Supplementary Figure S3** Chlorogenic acid contents (a) and transcript levels of *CsMYBF1* (b) in flesh tissues of the wild type and T_0_ transgenic tomato fruits.


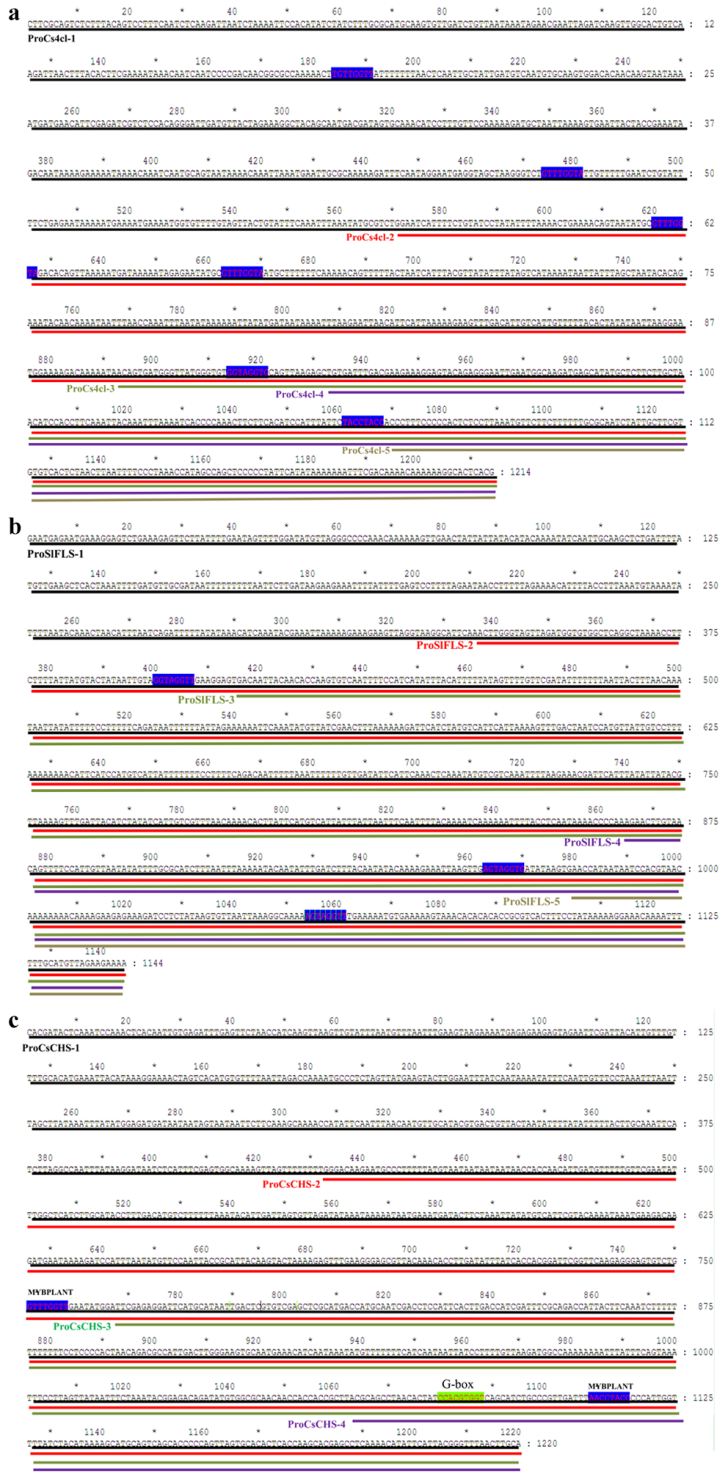


**Supplementary Figure S4** Detailed schematic diagram of various DNA fragments of the three selected promoters inked to the firefly luciferase reporter. Promoter sequences of *Cs4CL* (a), *SlFLS* (b), *CsCHS* (c).
